# Low-dose paclitaxel ameliorates fibrosis in the remnant kidney model by down-regulating miR-192

**DOI:** 10.1002/path.2961

**Published:** 2011-08-24

**Authors:** Lin Sun, Dongshan Zhang, Fuyou Liu, Xudong Xiang, Guanghui Ling, Li Xiao, Yinghong Liu, Xuejing Zhu, Ming Zhan, Yeyi Yang, Vinay K Kondeti, Yashpal S Kanwar

**Affiliations:** 1 Department of Nephrology, Second Xiangya Hospital, Central South UniversityChangsha, Hunan, People's Republic of China; 2 Emergency Department, Second Xiangya Hospital, Central South UniversityChangsha, Hunan, People's Republic of China; 3 Department of Pathology and Medicine, Northwestern UniversityChicago, IL, USA

**Keywords:** TGF-β1, tubulointerstitial fibrosis, microtubule, paclitaxel, Smad

## Abstract

Transforming growth factor (TGF)-β has been shown to play a central role in the development of tubulointerstitial fibrosis, which can be corrected via treatment with paclitaxel. The biology of microRNA (miR) can be modulated by paclitaxel. We hypothesized that paclitaxel may attenuate renal fibrosis in a rat model of remnant kidney disease by inhibiting TGF-β induced-miRs. Rats in groups of 12 were subjected to 5/6 nephrectomy and received low-dose intraperitoneal injection of paclitaxel. Renal functions were assessed at 8 weeks. The TGF-β signalling cascade and ECM proteins were evaluated by real-time polymerase chain reaction (TRT–PCR) and immunofluorescence microscopy. Animals with remnant kidneys developed hypertension, which was not relieved with paclitaxel treatment. However, paclitaxel treatment resulted in dampening the proteinuric response, reduction in serum BUN, creatinine levels and urine protein : creatinine ratio and normalization of creatinine clearance. These effects were accompanied by the inhibition of Smad2/3 activation, attenuation of renal fibrosis and normalization of integrin-linked kinase (ILK), COL(I)A1, COL(IV)A2 and α-SMA expression. Also, paclitaxel down-regulated the expression of miR-192, miR-217 and miR -377, while miR-15 was up-regulated in the remnant kidney. *In vitro*, in tubular epithelial cells (NRK-52E), paclitaxel also inhibited TGF-β1-induced Smad2/3 activation and normalized ILK, COL(I)A1, COL(IV)A2 and α-SMA expression. Furthermore, ChIP analyses indicated that Taxol suppressed Smad3-mediated miR-192 transcriptional activity. Over-expression of miR-192 in NRK-52E mimicked the changes seen in the remnant kidney, while inclusion of miR-192 inhibitor in the culture medium blocked TGF-β1-induced COL(I)A1 and COL(IV)A2 expression, while ILK and α-SMA were unaffected. These data suggest that low-dose paclitaxel ameliorates renal fibrosis via modulating miR-192 pathobiology and TGF-β/Smad signalling. Copyright © 2011 Pathological Society of Great Britain and Ireland. Published by John Wiley & Sons, Ltd.

## Introduction

Renal fibrosis, affecting either the glomerular or the tubulointerstitial compartment, is a common sequel of diverse chronic renal diseases. Tubulointerstitial fibrosis is a progressive process that ultimately leads to end-stage renal disease (ESRD), requiring dialysis or kidney transplantation 1. Among many factors, transforming growth factor-β (TGF-β) is a major well-characterized pro-fibrogenic cytokine whose expression is up-regulated in animal models of fibrosis, including in the remnant kidney model and also in human counterparts 2. It is believed that TGF-β modulates the transition of renal tubular epithelial cells to myofibroblasts and the latter apparently synthesize excessive amounts of extracellular matrix (ECM), thus leading to renal fibrosis 3–5. This is supported by the fact that blockade of TGF-β with the neutralizing antibody, antisense-oligo, decorin or siRNA attenuates renal scarring 6–9.

MicroRNAs (miRs) are short non-coding RNAs of 22 nt that have recently been shown to play an important role in mammalian gene expression 10. The miRs induce post-transcriptional gene repression by blocking protein translation (by binding to the 3′ UTR of their target genes) or by inducing mRNA degradation, and thus play a central role in various physiological and pathological processes. Recent evidence suggests that miRs may regulate the expression of key genes relevant to neoplastic processes and potentially other diseases as well 11–13. In kidney, miRs have been reported to play a role in podocyte development 14–16, the pathogenesis of diabetic nephropathy 17–19 and polycystic kidney disease 20. miR-192, miR-217 and miR-377 have been described to be up-regulated in diabetic mouse and mesangial cells treated with TGF-β or exposure to high-glucose ambience 17–19. Although it seems that the biology of miRs and TGF-β are interlinked, the effects of renal miRs may be cell type-specific, in contrast to TGF-β, which exerts its effect on a wide variety of cells. TGF-β induces ECM-related gene expression through a series of events following activation of its receptor. Activated TGF-β receptors stimulate the phosphorylation of receptor-regulated Smad2 and Smad3 proteins (R-Smads), which in turn form complexes with Smad4. This complex translocates from the cytoplasm into the nucleus, where the Smads regulate the transcription of target genes and miRs expression. Inhibitory Smad7 acts in an opposing manner to the R-Smads, and down-regulates TGF-β signalling 21,22. Some previous studies have shown that endogenous Smad-2, Smad-3 and Smad-4 bind to microtubules in several cell lines, and the binding provides a negative regulatory mechanism to modulate TGF-β activity 23. Disruption of the microtubule network by chemical agents, such as nocodazole and colchicine, leads to ligand-independent Smad nuclear accumulation and transcription of TGF-β-responsive genes 24.

Paclitaxel is an anticancer agent which, by stabilizing polymerized microtubules and maintaining microtubular assembly, arrests the cell cycle in the G_0_–G_1_ and G_2_–M phases and induces cell death 25,26. Prolonged chemotherapeutic treatment with paclitaxel has been associated with scleroderma-like changes or pulmonary fibrosis in some patients. It is noteworthy that inhibition of tumour cell proliferation can be achieved only by the administration of high dosages of paclitaxel. The inhibition of TGF-β–Smad signalling, however, can be accomplished with a very low dose of paclitaxel, which would minimally affect cell proliferation and other cellular activities. Interestingly, low-dose paclitaxel has been shown to inhibit collagen-induced arthritis, hepatic fibrosis and fibrosis associated with systemic sclerosis in SCID mice 26–28. Along similar lines, we previously reported that the low-dose administration of paclitaxel in a rat unilateral ureteral obstruction (UUO) model significantly reduces tubulointerstitial fibrosis 29. However, the molecular mechanism(s) by which paclitaxel inhibits renal fibrosis and ECM genes' transcription/translation remains to be clearly defined. In this regard, some of the literature reports suggest that miRs play a significant role in cytokine responses induced by paclitaxel. Murine macrophages incubated with paclitaxel had significantly increased expression of miR-155, miR-147, miR-146a and miR-132 30. In view of the above literature information, studies were initiated to assess whether stabilization of microtubules with low-dose paclitaxel (Taxol) is able to block TGF-β–Smad signalling and inhibit miRs expression, and thereby attenuate progressive renal injury in the 5/6 nephrectomized rat model.

## Materials and methods

### Animals

Animal experiments were performed in accordance with the regulations set by the Institutional Committee for the Care and Use of Laboratory Animals. Adult male Wistar rats (250–300 g body weight) were purchased from Shanghai Experimental Animal Centre of the Chinese Academy of Sciences, Shanghai, China. The animals were housed at 22 °C on a 12 hour light–dark cycle and were allowed free access to food and water.

### Animal model

The animals were anaesthetized with an intraperitoneal (i.p.) injection of freshly prepared Avertin. For generation of the remnant kidney model, 5/6 nephrectomy was performed, as described previously 31. Briefly, animals underwent subtotal nephrectomy involving right subcapsular nephrectomy and infarction of approximately two-thirds of the left kidney by ligation of the posterior and one or two anterior extrarenal branches of the renal artery. Four groups of rats, comprising 12 animals each (total = 48), were used: (I) Sham group—on the day of operation these rats received i.p. injection of phosphate buffer saline (PBS) and twice weekly thereafter; (II) Sham + Taxol group—the rats received an i.p. injection of paclitaxel (Taxol, Sigma, St. Louis, MO, USA) at a dose of 0.3 mg/kg twice weekly; (III) Rem group—the rats received and i.p. injection of PBS and underwent subtotal nephrectomy; (IV) Rem + Taxol group—the rats, in addition those in group III, received an i.p. injection of Taxol. All rats were euthanized at 8 weeks following surgery. Rat serum was collected at day 0 and at 4 and 8 weeks for creatinine determination. and 24 h urine samples were collected from the metabolic cages at day 0 and weekly thereafter for the determination of protein and creatinine excretion. At 8 weeks, the kidneys were harvested for various biochemical and morphological studies. All procedures were performed in accordance with institutional guidelines for animal care.

### Cell culture

#### Experimental protocol 1

Rat tubular epithelial cells (NRK-52E) from ATCC were cultured in Dulbecco's modified Eagle's medium (DMEM)–F12 medium supplemented with 10% fetal bovine serum (FBS). At 70% confluency the cells were transferred into complete medium containing 1% FBS and culture maintained in an atmosphere of 5% CO_2_/95% air at 37 °C. Four groups of experiments were performed: the Mock control group (treated only with DMEM); the Taxol group (200 nm paclitaxel was added to the cell culture), the TGF-β1 group [5 ng/ml recombinant human TGF-β1 (R&D Systems, Minneapolis, MN, USA) was added to the cell culture], and the TGF-β1 + Taxol group. After 48 h of treatment, the cells were processed for various studies. The experiments *in vitro* were basically designed to mimic the *in vivo* conditions, since increased expression of TGF-β1 has been well described in the remnant kidney model. All the experiments were carried out in quadruplicate.

#### Experimental protocol 2

The cells were seeded in a 24-well plate at a density of 10^5^ cells/well, using DMEM/F12 medium containing 5% fetal calf serum (FCS). By employing Lipofectamine 2000, subconfluent cells were then transfected with four different miRs: hsa-miR-192 mimic (50 nm); negative control (miR-neg; Sigma); antisense-miR-192 (400 nm); or control antisense-miR (Genepharma, Shanghai, China). After 24 h of transfection, the culture media were replaced with fresh medium and the cells were allowed to achieve 80% confluency, following which the cells were maintained in a serum-free medium overnight. They were then treated with either 0.1% BSA (control) or TGF-β1 (5 ng/ml) for another 24 h. Cell extracts were prepared from various experiments for real-time PCR or western blot analyses and immunofluorescence studies.

### Measurements of protein and creatinine

The rats were placed in individual metabolic cages and 24 h urine collection was carried out. The urine samples were centrifuged at 2000 × *g* for 5 min and the supernatants were saved. Urine albumin was measured by enzyme-linked immunosorbent assay (ELISA; Bethyl Laboratories, Montgomery, TX, USA). Urine albumin excretion (UAE) was normalized with creatinine excretion and expressed per mg creatinine. Creatinine levels in the urine and serum were measured using the QuantiChrom Creatinine Assay Kit (BioAssay Systems, Hayward, CA, USA).

### Blood pressure measurements

Systolic blood pressure (SBP) was measured in conscious rats by use of a non-invasive computerized tail-cuff system (Model-1231, IIT Inc), as described previously 32.

### Morphological studies

Kidney tissues were fixed with 4% buffered paraformaldehyde, embedded in paraffin, and 4 µm thick sections were prepared. The sections were then stained with periodic acid–Schiff (PAS) and Masson's trichrome 33. The mean glomerular cross-sectional area (glomerulosclerosis index, GSI) was determined in 50 glomerular sections from each rat 33. All analyses were performed in a blind manner. Segmental and complete glomerular sclerosis were analysed using a semiquantitative scoring system in the range 0–4 (0, no glomerulosclerosis; 1, < 25% of glomerular area affected; 2, 25–50% affected; 3, 50–75% affected; 4, 75–100% affected); at least 50 glomeruli were evaluated under × 400 magnification and the results were averaged. The tubulointerstitial injury score was estimated. The GSI for each rat was calculated as a mean value obtained from 50 glomeruli. The tubulointerstitial injury score was estimated based on the number of tubule dilatations, the distortion of the tubular basement membranes, and atrophy in the range 0–3 [0, none (<5%); 1, mild (5–25%); 2, moderate (25–50%); 3, severe (>50%)]. More than 10 consecutive fields were examined at a magnification of × 400 34. The score index in each rat was expressed as a mean value of all scores obtained.

### Real-time polymerase chain reaction

Total RNA was isolated using the High Pure RNA Isolation Kit (Roche, Switzerland) according to the manufacturer's instructions. Contaminated DNA was removed by treating the samples with RNAase-free DNAase I (Promega, Madison, WI, USA). First-strand cDNAs were generated using a superscript VILO cDNA synthesis kit (Invitrogen). Real-time PCR was performed using a Bio-Rad (Hercules, CA) IQ SYBR Green Supermix with Opticon (MJ Research Inc., Waltham, MA, USA). The primer sets used for various genes were as follows:

- *Smad2*: forward 5′-TCACAGCCATCATGAGCT CAAGG-3′, reverse 5′-TGTGACGCATGGAA GGTCTCTC-3′.
- *Smad3*: forward 5′-AGCACACAATAACTTGG ACC-3′, reverse 5′-TAAGACACACTGGAACA GCGGATG-3′.
- *Integrin-linked kinase* (*ILK*): forward 5′-CCGCT GGCAGGG CAATGACATT-3′, reverse 5′-GGG GGAGCCTGGCAAGCACCTA-3′.
- α*2-Smooth muscle actin* (α*2-SMA*): forward 5′-ACTGGGACGACATGGAAAAG-3′, reverse 5′-CATCTCCAGAGTCCAGCACA-3′.
- *Collagen type I* α*1* (*COL1A1*): forward 5′-GAG CGGAGAGTA CTGGATCG-3′, reverse 5′-TAC TCGAACGGGAATCCATC-3′.
- *Collagen type IV* α*2* (*COL4A2*): forward 5′-AC ACTGTGGACTTACCAGG-3′, reverse 5′-CC AGGAAATCCAATGTCACC-3′.

All samples were subjected to RT–PCR along with the housekeeping gene *GAPDH*, having the following primer sequences:

1. *GADPH*: forward 5′-TGCTGAGTATGTCGTGG AGTCTA-3′, reverse 5′-AGTGGGAGTTGCTG TTGAAATC-3′

as an internal standard control. The PCR conditions were as follows: 94 °C for 2 min, followed by 40 cycles of (94 °C for 15 s, 58 °C for 30 s and 72 °C for 30 s) and a final extension at 72 °C for 10 min.

### miRNA extraction and microarray analysis

**Figure 5:**
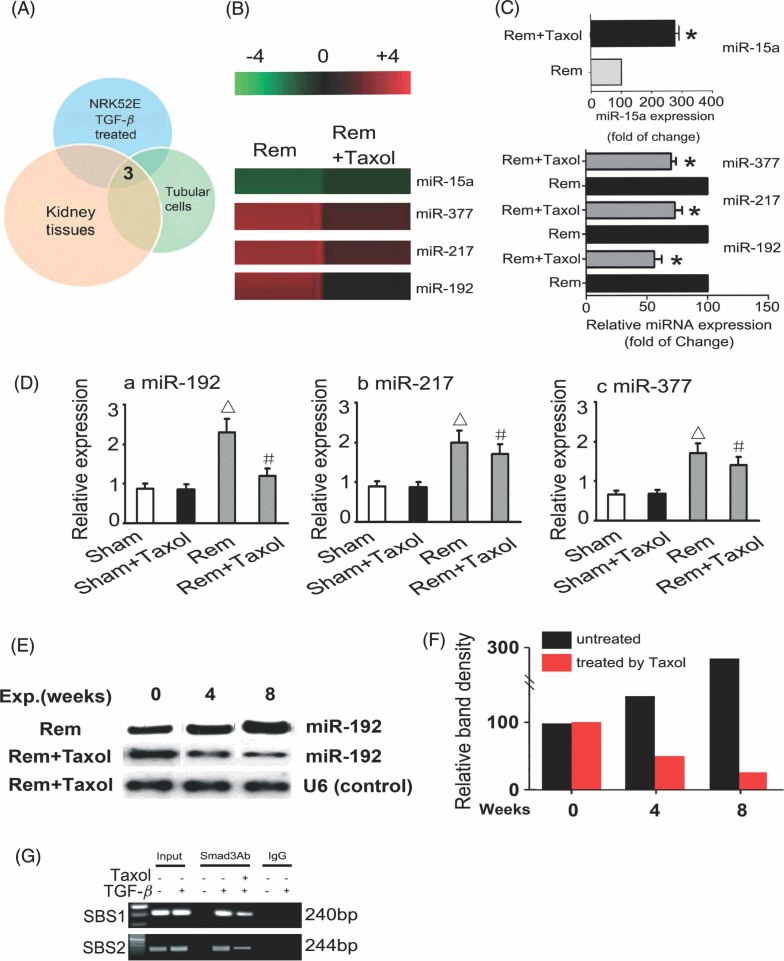
Expression of miRNA in the rat remnant kidney, isolated tubular cells and TGF-β1-treated NRK52E cells. (A) Comparative microarray analysis indicated that three miRNAs were up-regulated in all the samples. (B) Log_2_ values of each Rem + Taxol/Rem pair of miRNA microarray signals were displayed in a heat map generated by a TIGR multi-experiment; red indicates up-regulation, green down-regulation and black no change. The bar code (−4 to + 4) at the top represents the colour scale of the log_2_ values. (C) Bar graph indicating fold changes in the expression of miRNAs (*miR-15a, miR-377, miR-217* and *miR-192*) in Rem + Taxol compared to that of the Rem group. Taxol treatment reduced the expression of three miRNAs, especially that of *miR192*, as assessed by microarray analyses. (D) Real-time PCR confirmed the increased *miR-192, miR-217* and *miR-337* expression in the rat remnant kidney; these expressions were significantly higher in Rem rats than in Sham animals (*p* < 0.05, *n* = 12), and the expression was notably reduced following Taxol treatment (*p* < 0.05, *n* = 12). No change was observed between the Sham and Sham + Taxol groups. (E) Northern blot analyses showed the time-course effect of Taxol on miR-192 expression levels in the kidney of the rat remnant model. miR-192 expression increased significantly at 4 and 8 weeks following surgery and was substantially reduced with Taxol treatment. (F) Densitometry analyses of northern blots from six independent experiments; each bar represents mean ± SEM for 12 animals. *p < 0.05 versus Rem group; ^Δ^*p* < 0.05 versus Sham group; ^#^*p* < 0.05 versus Rem group (*n* = 12). (G) ChIP assays for Smad3 were performed with chromatin material isolated from NRK52E cells treated with TGF-β1. Precipitated DNA was amplified with oligonucleotides spanning regions of the potential Smad binding sites 1 and 2 (SBS1 and SBS2); total inputs are indicated. The antibody against Smad3 immunoprecipitated the DNA fragments from NRK52E cells containing the potential SBS1 and SBS2, and their bindings were suppressed by Taxol

miRNAs were extracted from isolated tubular cells or renal cortices enriched with tubules or NRK52E tubular cell lines from different experimental variables (Figure 5), using a miRNwasy Mini Kit (Qiagen, Valencia, CA, USA). miRNA microarray procedures were performed at Beijing Capital Biological Corporation. Briefly, isolated miRNA was covalently linked with either Cy3 (green channel) or Cy5 (red channel). Pairs of labelled samples were hybridized to dual-channel microarrays. A double-channel laser fluorescence scanner was used for microarray analyses, and the data were analysed as described previously 35. Successful array submission was made to MIAMExpress (http://www.ebi.ac.uk/miamexpress), array design name ‘Taxol-treatment-mechanism’.

### Northern blot analysis of miRNA

Total RNA was extracted from kidney cortices using Trizol (Invitrogen). Low-molecular weight RNA was subsequently isolated by precipitation in PEG 8000/ NaCl, as previously described 36. Low-molecular weight RNA (50 µg) was run on a denaturing 10% polyacrylamide gel, transferred to PVDF membrane (Amersham/GE Healthcare, Piscataway, NJ, USA), subjected to UV light irradiation for 4 min and baked at 80 °C for 1 h. The locked nucleic acid (LNA)-modified miR-192 oligonucleotide probe (5′-CTGACCTATGAATTGACAGCC-3′) was purchased from Exiquon (Woburn, MA, USA). The probe was 5′-end-labelled with (γ-^32^P) ATP (Perkin-Elmer Life Sciences). Signals were quantified using National Institutes of Health Image J version 1.42q software.

### Detection of miRNA expression

Total RNA was extracted from kidney. The RNA samples were reverse-transcribed to cDNA using the mirVanat qRT–PCR miRNA detection kit (Ambion). Real-time PCR was carried out using SYBR Premix EXTaqTM (Takara), following the manufacturer's instructions. The miR-192, miR-217, miR-337 and miR-15a primer sets were purchased from RiBi Biological Corporation (Guangzhou, China). The input was normalized with U6 smRNA. Data were shown as fold change (2^−ΔΔCt^) and analysed using Opticon Monitor Analysis software v2.02 (MJ Research, Waltham, MA, USA).

### Immunofluorescence and immunohistochemistry

Cells were grown on coverslips, washed twice with PBS, fixed in 4% paraformaldehyde for 20 min, permeabilized using 0.1% Triton X-100 and incubated in a blocking buffer (1% BSA, 0.25% Triton X-100 in PBS, pH 7.4). Primary and secondary antibodies were diluted in a blocking buffer and incubated with cells overnight at 4 °C. After DAPI staining the cover slips, including the cells, were mounted onto glass slides after placing a drop of Prolog Gold Antifade reagent. The cells were then viewed using a fluorescence microscope. For tissue immunofluorescence studies, cryostat sections (4 µm thick) were fixed in cold acetone and then incubated with mouse monoclonal anti-COL(I)A1 (Abcam) or rabbit polyclonal anti-COL(IV)A2 (Abcam) antibody for 1 h, followed by another incubation with FITC-labelled anti-mouse or rabbit IgG (Biosource International, Camarillo, CA, USA). Immunohistochemical analyses were carried out by using rabbit anti-phospho-Smad2/3 (Santa Cruz Biotechnology) and mouse monoclonal anti-α-SMA (Santa Cruz). The sections were deparaffinized and quenched with 3% H_2_O_2_ for 10 min to block endogenous peroxidase and then washed in PBS. They were incubated with various primary antibodies, as listed above, for 1 h and then with biotinylated secondary antibody, followed by ABC reagent treatment as recommended by the vendor. Colour development was achieved by incubating the sections with diaminobenzidine (DAB) as substrate. The slides were counterstained with Mayer's haematoxylin. Preincubation of the primary antibody with specific blocking peptides or substitution of the primary antibody with an irrelevant IgG served as negative controls. The slides were examined using an Olympus microscope equipped with UV epi-illumination. The intensity of fluorescence was analysed by image analysis software (Path QC, Logene Biological Medical Engineering Co. Ltd).

### Western blotting

Western blot analyses were carried out as described previously 33. Briefly, samples (20 µg protein) were subjected to SDS–PAGE. After transfer of proteins onto the nitrocellulose membrane (Amersham), the blots were probed with the following antibodies: mouse monoclonal anti-α-SMA (Santa Cruz; 1 : 1000 dilution), -COL(I)A1 (Abcam; 1 : 1000 dilution), rabbit polyclonal anti-phospho-Smad2/3 (Santa Cruz Biotechnology; 1 : 1000 dilution), -COL(IV)A2 (Abcam; 1 : 1000 dilution) and -ILK (Santa Cruz; 1 : 1000 dilution). A peroxidase-conjugated goat anti-mouse IgG (1 : 20 000 dilution) was used as a secondary antibody. Pig anti-rabbit IgG or rabbit anti-goat in PBS containing 1% normal goat serum and 1% FCS and β-actin were used as internal controls.

### ChIP analysis

Chromatin immunoprecipitation (ChIP) analysis was performed using a transcription factor ChIP kit according to the manufacturer's instructions. In brief, cells were cross-linked with 1% formaldehyde for 10 min at 37 °C, quenched with glycine and then sonicated, using a Bioruptor (Diagenode, Liège, Belgium) to generate 300–600 bp DNA fragments. Immunoprecipitation was performed with the antibody directed against Smad3 (Upstate) and IgG was used as a control. Precipitated DNAs were subjected to PCR analysis using specific primers as follows: for Smad binding site 1 (SBS1), 5′-AGCCAAGTGTCTCTCCGTGT-3′ and 5′–GGTGGCAGGAGGTGTGTACT-3′; for Smad binding site 2 (SBS2), 5′-CACTGTCCCAACATGCT CAC-3′ and 5′-GGTAGCAGGGAGAAGCAATG-3′. The reaction mix consisted of 1.5 µl template, 5.0 µl 2× Bio-RadiQSYBR Green supermix, 0.3 µl forward primer, 0.3 µl reverse primer and 2.9 µl double-distilled H_2_O. qPCR was performed as described above for quantitative real-time PCR.

### Statistical analysis

Data were expressed as mean ± SD and one-way analysis of variance (ANOVA) was carried out. *p* < 0.05 was considered statistically significant.

## Results

### Biochemical data

**Figure 1:**
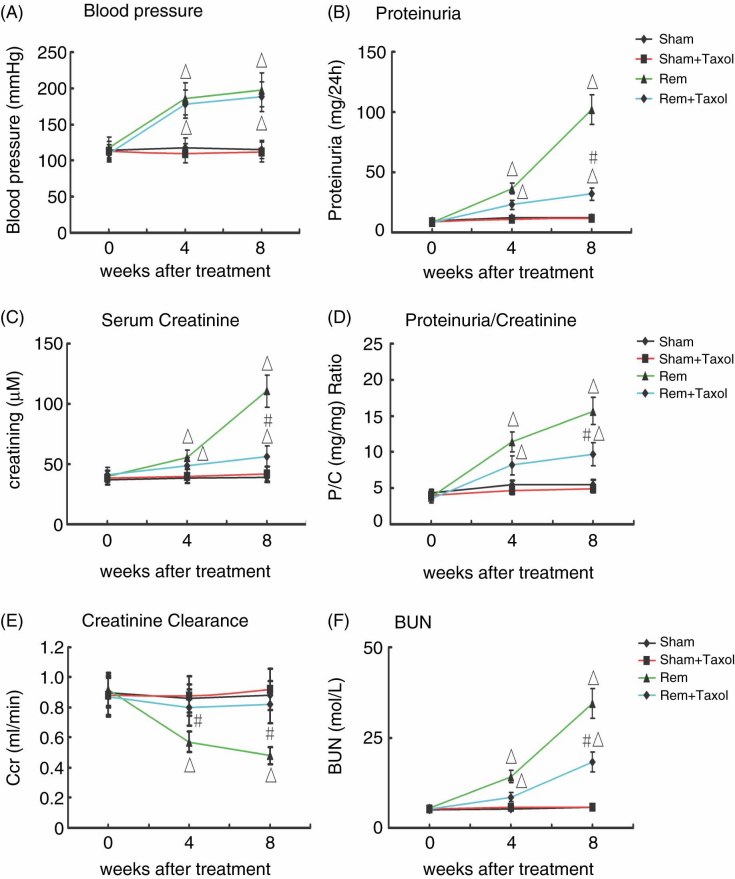
Effect of paclitaxel (Taxol) on blood pressure and renal functions of rats with remnant (Rem) kidney. (A) Systolic blood pressure; (B) 24 h urinary protein excretion; (C) serum creatinine; (D) protein : creatinine ratio; (E) creatinine clearance; (F) BUN. All the renal functional parameters were almost normalized with Taxol treatment in the Rem group; however, the blood pressure was unaffected by Taxol treatment. Data represent mean ± SEM for groups of 12 rats treated with Taxol. ^Δ^*p* < 0.05 versus Sham or Sham + Taxol groups; ^#^*p* < 0.05 versus Rem group (*n* = 12)

Rats that had undergone subtotal nephrectomy developed hypertension at the end of the second week and it was not relieved by the paclitaxel (Taxol) treatment (Figure 1A). These rats (designated the Rem group) developed progressive renal injury, as reflected by an increase in 24 h urinary protein excretion (Figure 1B), rise in serum creatinine (Figure 1C), proteinuria : creatinine ratio (Figure 1D) and blood urea nitrogen (BUN) (Figure 1F), and decline in creatinine clearance (Figure 1E). In contrast, paclitaxel treatment blunted the proteinuric response, rise in serum creatinine, protein : creatinine ratio and BUN (Figure 1B–D, F; *p* < 0.05), and prevented the drop in creatinine clearance after 5/6 nephrectomy (Figure 1E; *p* < 0.05).

### Effects of paclitaxel on morphology in the remnant kidney

**Figure 2:**
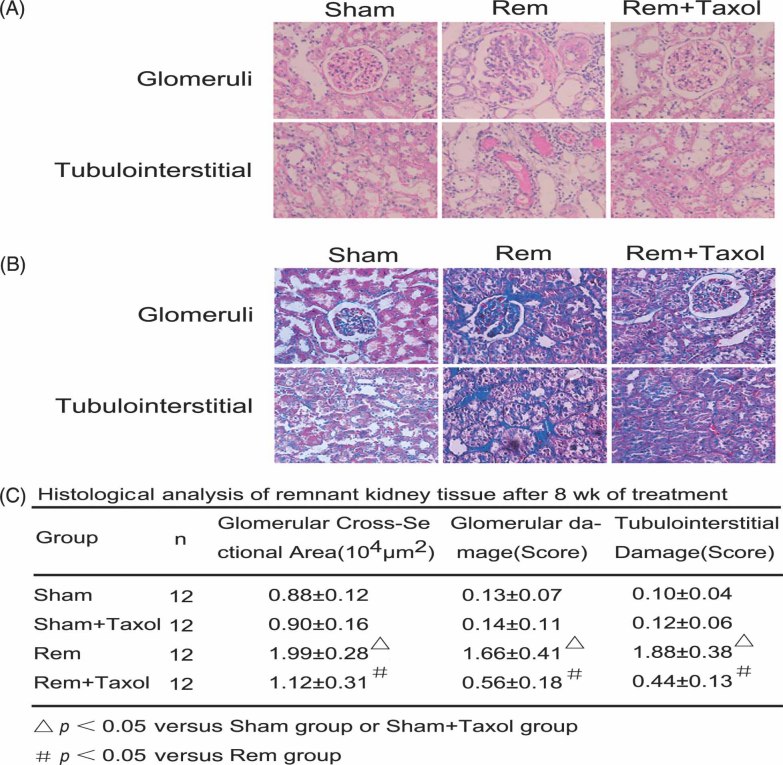
Effect of Taxol on renal morphology of rat remnant kidney. Kidney sections were stained with PAS (A) and Masson's trichrome (B). There were remarkable changes in both glomerular and tubulointerstitial compartments in the remnant kidney, which were notably reduced by the Taxol treatment. (C) Morphometric analyses confirmed the morphological changes seen in various groups. Magnification, × 400. ^Δ^*p* < 0.05 versus Sham or Sham + Taxol groups; ^#^*p* < 0.05 versus Rem group (*n* = 12)

At 8 weeks after surgery, notable glomerulosclerosis and tubulointerstitial fibrosis was observed, as assessed by PAS and Masson's trichrome staining (Figure 2A, B). In the 5/6 nephrectomy kidneys the glomerulosclerosis index (GSI) was ∼11-fold higher compared to Sham control kidneys and interstitial expansion was also a prominent feature. Treatment with Taxol significantly reduced the progression of glomerular injury and interstitial expansion. The glomerular cross-sectional area was notably reduced in the Rem + Taxol group compared to the Rem group (Figure 2C, 1.99 → 1.12). Also, tubulointerstitial fibrosis was significantly reduced by Taxol treatment (Figure 2C, 1.86 → 0.44).

### Inhibition of Smad2/3 activation and reduction of ECM expression following Taxol treatment in the remnant rat kidney

**Figure 3:**
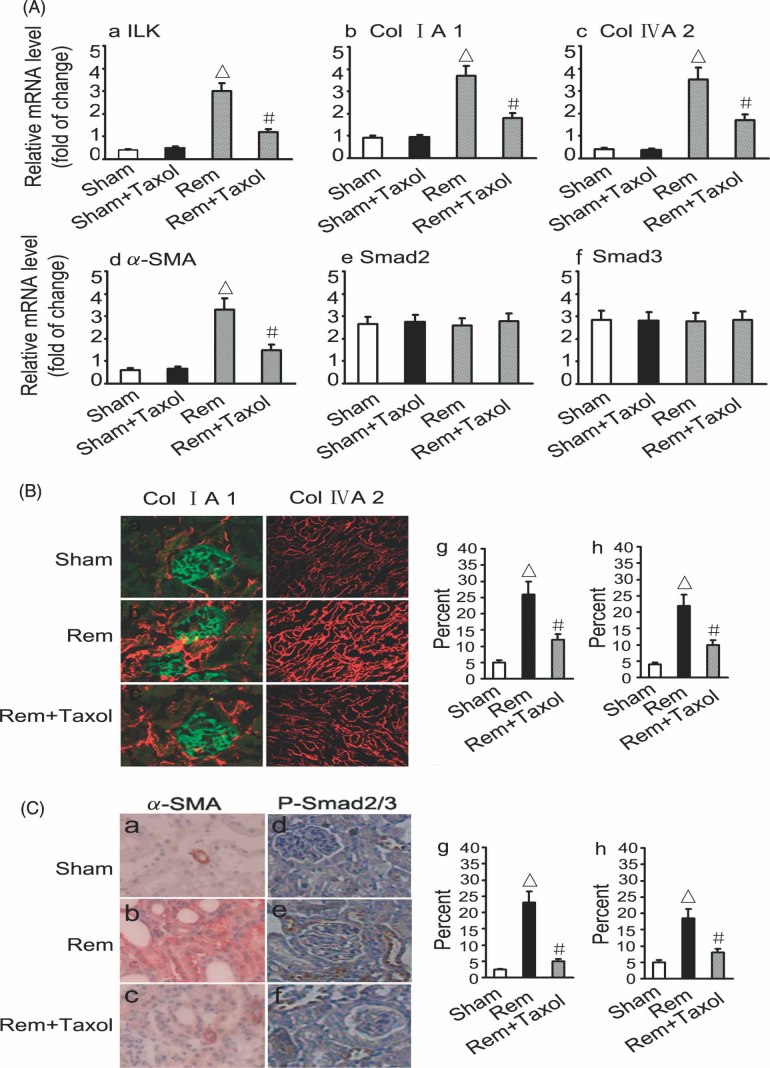
Effect of Taxol on ILK, collagen, phosphorylated Smad2/3 (p-Smad) and α-SMA expression in the rat remnant kidney. (A) Real-time PCR analyses revealed that Taxol inhibited the expression of ILK, COL(I)A1, COL(IV)A2 and α-SMA in the rat remnant kidney (*p* < 0.05, *n* = 12). No differences in *Smad2* and *Smad3* mRNA expression were observed in various experimental groups. (B) Immunofluorescence microscopy showed that expression of COL(I)A1 and COL(IV)A2 was significantly increased in the remnant kidney (*p* < 0.05, *n* = 12) and notably reduced with Taxol treatment (*p* < 0.05, *n* = 12). (C) Immunohistochemistry showed that expression of p-Smad2/3 and α-SMA was significantly increased in the remnant kidney (*p* < 0.05, *n* = 12) and notably reduced with Taxol treatment (*p* < 0.05, *n* = 12). Data of each bar represents mean ± SEM for 12 animals. ^Δ^*p* < 0.05 versus Sham group (*n* = 12); ^#^*p* < 0.05 versus Rem group. Magnification, × 400

The effects of Taxol on mRNA and protein expression of ILK, COL(I)A1, COL(IV)A2 and α-SMA were determined by real-time reverse transcription–polymerase chain reaction (TRT–PCR) and immunofluorescence microscopy. TRT–PCR analyses showed that Taxol has no effect on ILK, COL(I)A1, COL(IV)A2 and α-SMA expression in Sham-operated rat kidneys, while it remarkably reduced their expression in the remnant kidney (Figure 3A). Taxol had no effect on *Smad2* and *Smad3* mRNA expression in Sham-operated or remnant kidneys. Immunofluorescence microscopy and immunohistochemistry revealed a notable reduction in the expression of Collagen I, Collagen IV, α-SMA and the phosphorylated forms of Smad2/3 with Taxol treatment in the remnant kidney (Figure 3B). The RT–PCR data are representative of six experiments.

### Taxol inhibits TGF-β1-induced expression of ILK, COL(I)A1, COL(IV)A2, α-SMA, and Smad2/3 phosphorylation in renal tubular epithelial cells

**Figure 4:**
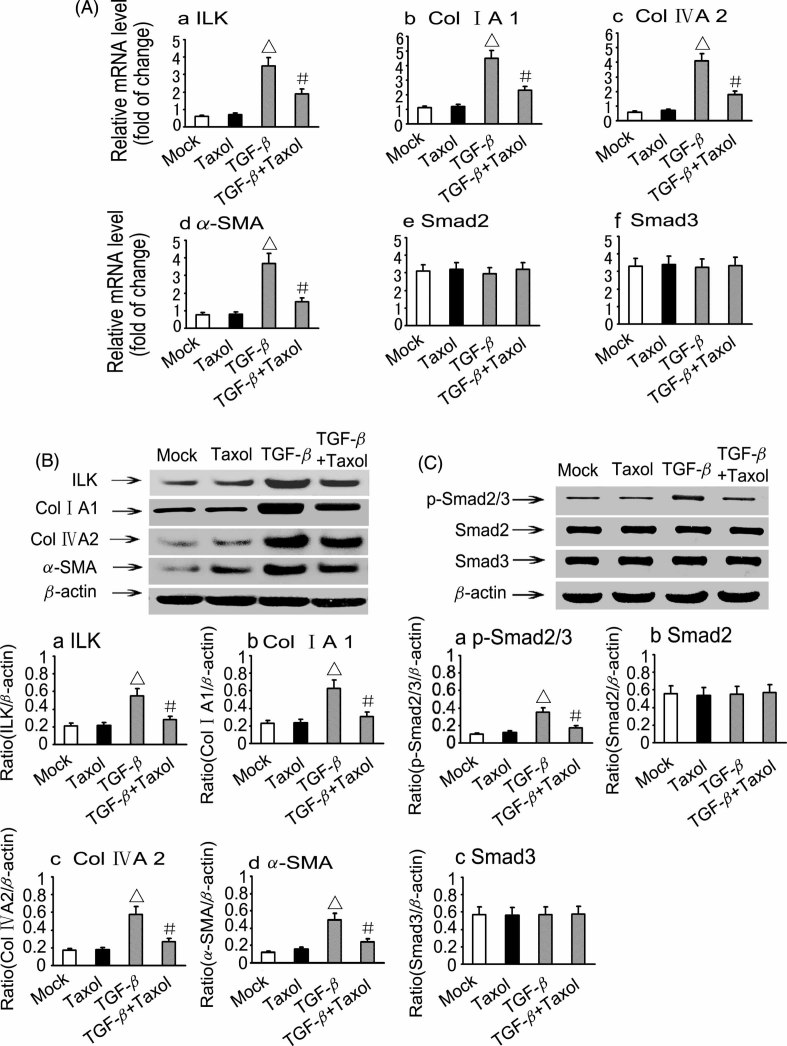
Effect of Taxol on ILK, Collagen, phosphorylated Smad2/3 (p-Smad), Smad2, Smad3 and α-SMA expression in NRK52E cells. (A) Real-time PCR analyses showed that *ILK, Collagen* and α*-SMA* were significantly higher in the TGF-β1 group compared to the Mock group (*p* < 0.05, *n* = 6). Treatment with Taxol caused a significant decrease in their expression in TGF-β1-treated NRK52E cells (*p* < 0.05, *n* = 6). *Smad2* and *Smad3* mRNA expression were unaltered in the Taxol and TGF-β1 + Taxol groups. (B, C) Western blot analyses showed that ILK, Collagen, p-Smad2/3 and α-SMA, but not total Smad2 or Smad3, expressions were significantly higher in the TGF-β1 group compared to the Mock group (*p* < 0.05, *n* = 6), while treatment with Taxol reduced their expression (*p* < 0.05, *n* = 6). ^Δ^*p* < 0.05 versus Mock or Taxol groups (*n* = 6); ^#^*p* < 0.05 versus TGF-β1 group

The effect of Taxol on mRNA and protein expression of ILK, COL(I)A1, COL(IV)A2, α-SMA, Smad2 and Smad3 were determined by TRT–PCR and western blot analyses. TRT–PCR analyses indicated that Taxol significantly reduced the expression of ILK, COL(I)A1, COL(IV)A2 and α-SMA in TGF-β1-treated NRK52E cells; however, no effect was seen on Smad2 and Smad3 (Figure 4A). Western blot analyses confirmed the results observed in the above gene expression following Taxol treatment in TGF-β1-stimulated NRK52E cells (Figure 4B). Also, Taxol had no effect on the protein expression of Smad2/3, but the phosphorylated form was significantly reduced (Figure 4C).

### Regulation miRNAs expression by Taxol

Comparative microarray analysis of intact kidney tissue (cortices from remnant kidney), isolated renal tubular epithelial cells and TGF-β1-treated (5 ng/ml) NRK52E cells revealed that the changes in expression were mainly confined to three miRNAs (Figure 5A). Taxol down-regulated miR-192, miR-217 and miR-377 in remnant kidney, while it up-regulated miR-15a by microarray (Figure 5B, C) and real-time PCR analyses (Figure 5D). Since the most remarkable changes were seen in miR-192, the studies were extended and its expression was also assessed by northern blot analyses in the remnant kidney. In the remnant kidney, the miR-192 expression increases notably by 4 weeks, and by 8 weeks a steep increase was observed. The expression was dramatically reduced with the Taxol treatment (Fig 5E, F). Furthermore, we used a ChIP assay to determine the interaction of Smad3 with the miR-192 promoter region in NRK52E cells. As shown in Figure 5G, the antibody directed against Smad3 immunoprecipitated the DNA fragments from NRK52E cells containing the potential binding sites of SBS1and SBS2, supporting the hypothesis that Smad3 can physically interact with the miR-192 promoter region, and their activities were remarkably suppressed by Taxol.

### Modulation of COL(I)A1 and COL(IV)A2 expression by miR-192 and TGF-β1 in NRK52E renal tubular epithelial cells

**Figure 6:**
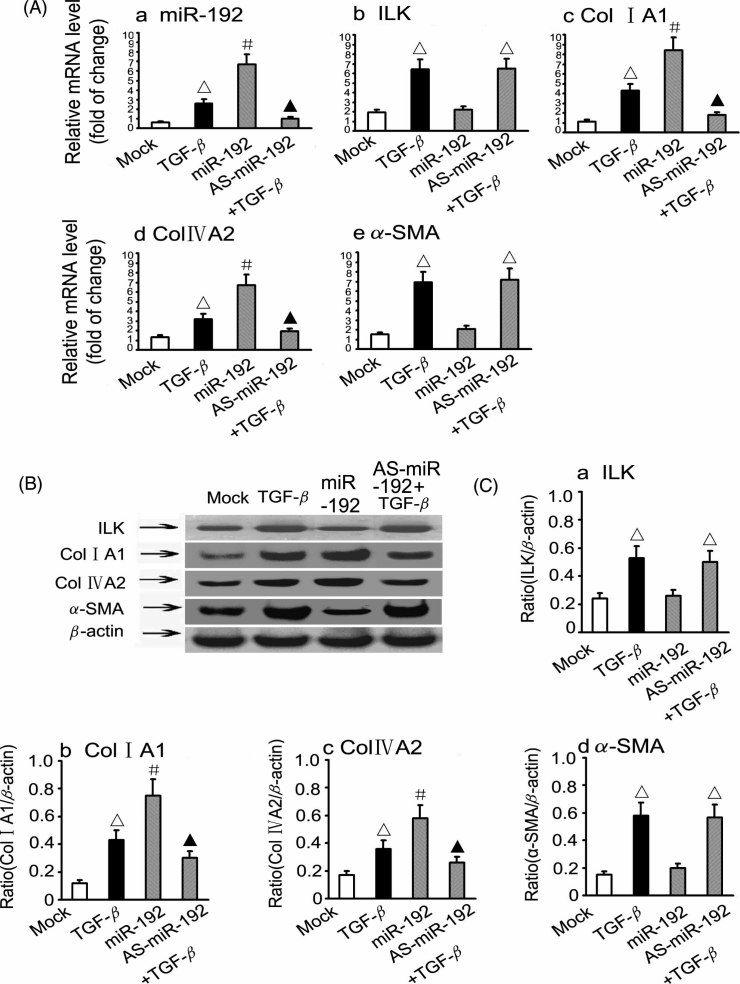
Effect of miR192 transfection on ILK, COL(I)A1, COL(IV)A2 and α-SMA expression in NRK52E cells. (A) Real-time PCR analyses revealed that expression of miR192, ILK, COL(I) A1, COL(IV)A2 and α-SMA were markedly increased in TGF-β1-treated NRK52E cells (*p* < 0.05, *n* = 6). Also, the expressions of COL(I)A1, COL(IV)A2 and miR192, but not of ILK and α-SMA, were increased in *miR-192*-transfected NRK52E cells compared to Mock (*p* < 0.05, *n* = 6). The effects accentuated by TGF-β1 were abolished with the AS-miR-192 treatment (*p* < 0.05, *n* = 6). (B, C) Western blot analyses displayed similar results; data were derived from six independent experiments. ^Δ^*p* < 0.05 versus Mock group; ^#^*p* < 0.05 versus TGF-β1 group; ^▴^*p* < 0.05 versus miR192 group (*n* = 6)

**Figure 7:**
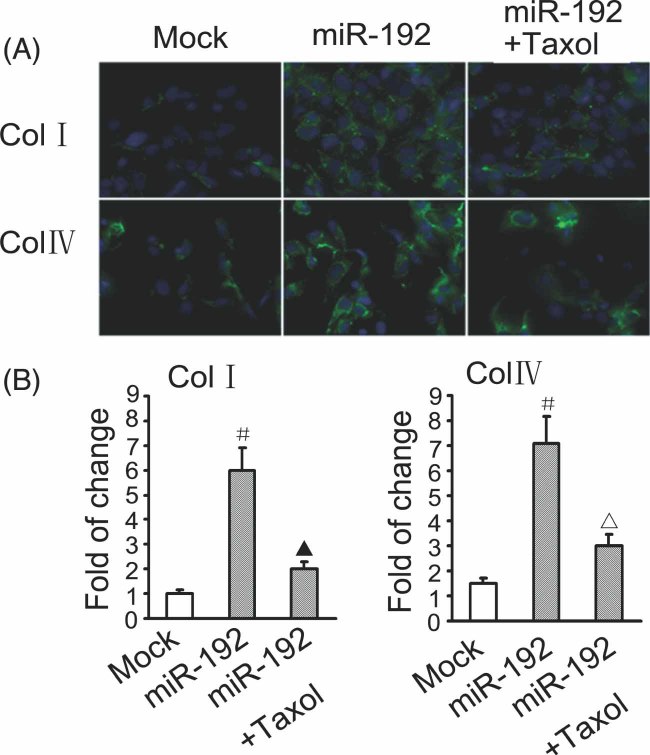
Effect of *miR192* transfection on COL(I)A1 and COL(IV)A2 expression in NRK52E cells, as assessed by immunofluorescence microscopy. The *miR-192*-transfected NRK52E cells had a significantly increased expression of both collagen I and collagen IV compared with the Mock group (*p* < 0.05, *n* = 6). Taxol treatment notably reduced the expression of both the ECM proteins, ie COL(I)A1 and COL(IV)A2. ^#^*p* < 0.05 versus Mock group (*n* = 6); ^▴^*p* < 0.05 versus miR192 group (*p* < 0.05, *n* = 6)

Treatment with TGF-β1 induced an increase in the expression of *ILK, COL(I)A1, COL(IV)A2* and α-*SMA* mRNA from the baseline (Mock) by TRT–PCR (Figure 6A). The miR-192 transfection led to a two- to three-fold higher expression of COL(I)A1 and COL(IV)A2 compared to the treatment with TGF-β1, while that of ILK and α-SMA were indistinguishable (Figure 6A). The transfection of AS-mIR-192 caused a remarkable reduction in the expression of COL(I)A1 and COL(IV)A2 in TGF-β1-treated cells, while that of ILK and α-SMA were unaltered. Western blot analyses confirmed the findings of the above gene expression studies (Figure 6B, C). In view of this, immunofluorescence studies were also carried out to assess the effect of Taxol. An increase in the cellular expression of COL(I)A1 and COL(IV)A2 was observed with the transfection of miR-192, and it was remarkably reduced with Taxol treatment (Figure 7A, B).

## Discussion

The data from this investigation suggest that low-dose Taxol ameliorates renal fibrosis with improvement in renal functions in the rat remnant kidney model by reducing collagen synthesis in the tubulointerstitial compartment. The central mechanism seems to be that Taxol interferes in the TGF-β1-induced downstream signalling events, which include blockade of Smad2/3 activation and inhibition of miRNA-192, with a net result of decreased expression of collagen both *in vitro* and *in vivo* systems.

The remnant kidney model has been widely used to study various pathogenetic mechanisms that lead to tubulointerstitial fibrosis and chronic kidney disease (CKD). In CKD, TGF-β1 has long been considered to be an important modulator of renal tubular biology, with increased collagen production that ultimately leads to fibrosis and scarring of the kidney 37,38. The fibrogenic effects of TGF-β1 are thought to be mediated via the Smad2/3 pathway, where Smad3 plays a critical role as a downstream signalling molecule 21. Loss of Smad3 activity has been shown to yield protection from radiation-induced fibrosis 39, bleomycin-induced pulmonary fibrosis 40 and tubulointerstitial fibrosis in the UUO model 41. In the orchestration of these events related to fibrosis, cellular microtubules (MTs) serve as negative regulators of TGF-β/Smad signalling. They form a complex with endogenous Smad2, Smad3 and Smad4, and thus sequester receptor-Smads (R-Smads) away from TGF-β receptor modulation 21. Conceivably, the disruption or dynamic instability of the cellular MT network leaves TGF-β uncomplexed and thus free to exert its fibrogenic effects. In this regard, stabilization of MTs with low-dose Taxol has been shown to dampen the exaggerated TGF-β signalling and thus block TGF-β-induced inhibition of myogenesis in C2C12 myoblasts 42,43. Similarly, Taxol has been reported to significantly suppress TGF-β/Smad activity and fibrosis in SCID mice and the rat UUO model 28,29. Along these lines, the present study provides evidence that low-dose Taxol suppresses TGF-β/Smad activity in the rat remnant kidney and TGF-β-induced ECM expression in tubular epithelial cells.

The finding that low-dose Taxol reduces the up-regulation of COL(I)A1, COL(IV)A2 and α-SMA in the rat remnant kidney without lowering the systemic blood pressure suggests that the reno-protective effect of paclitaxel is independent of angiotension II activity, etc., and is solely directed at ECM synthesis. It is conceivable that stabilization of the MT network maintains appropriate inside–out or outside–in signals and proper cellular homeostasis, so that excessive synthesis of ECM does not occur. In inside–out or outside–in signalling the ILKs play a crucial role, since these intracellular serine/threonine protein kinases are known to regulate cell adhesion, survival and epithelial–mesenchymal transition (EMT) 44. Increasing evidence suggests that inhibition of ILKs attenuates renal interstitial fibrosis, where ILK induction by TGF-β1 is clearly dependent upon Smad signalling in tubular epithelial cells 45. The subject matter of EMT is somewhat controversial, however; the up-regulation of α-SMA and down-regulation by Taxol, with concomitant decrease in the synthesis of collagen, suggest that transformation of tubular cells to myofibroblasts, reminiscent of EMT, may occur at least *in vitro* where TGF-β1 effects are nullified by Taxol.

To further investigate the mechanisms by which Taxol reduces fibrosis in the rat remnant kidney model, microarray analyses were carried out using renal cortices, isolated renal tubular epithelial cell and TGF-β1-treated NRK52E cells. The expressions of three miRNAs, miR-217, miR-192 and miR-377, were found to be consistently high, and they were decreased with low-dose paclitaxel treatment in all three different biological samples, ie cortices, isolated tubular cells and NRK52E cells. The miR-217 is known to be involved in increased collagen production and the progression of diabetic nephropathy through the down-regulation of PTEN and the subsequent activation of Akt kinase, and similar Akt up-regulation can be achieved by TGF-β-induced miR-192 18. miR-377 is thought to regulate the expression of fibronectin, another ECM protein that is up-regulated in diabetic nephropathy. Expression of miR-377 has been found to be up-regulated in various mouse models of diabetic nephropathy and in cultured human and mouse mesangial cells subjected to a high-glucose ambience or treated with TGF-β1 19. The role of miR-192 in kidney diseases is still controversial. Kato *et al* 17 described its up-regulation in the glomeruli of type 1 and type 2 diabetic mice and in cultured mesangial cells treated with TGF-β1. Kato *et al* and Chung *et al* have reported that miR-192 was up-regulated in cultured mesangial and tubular cells treated with TGF-β1 18,46. They also reported that TGF-β1-induced miR-192 up-regulation in mesangial cells increased the expression of COL(I)A2 by down-regulating Zeb2, an E-box repressor 17.

The findings of Wang *et al* and Krupa *et al* 47,48 in rat proximal tubular and mesangial cells are contrary to the results reported by Kato *et al* and Chung *et al* 18,46. Such differences may be related to the different culture conditions used. Kato *et al* used serum-deprived cells to establish a baseline for measuring the effect of TGF-β. They observed a 70% drop in miR-192 expression with serum deprivation, which was essentially reversed by TGF-β. In the Wang *et al* experiments, under reduced serum conditions the TGF-β1 treatment resulted in a decrease rather than increase in miR-192 levels. Kato *et al* and Chung *et al* 18,46 also reported an increase in miR-192 in the diabetic kidney, UUO and the rat 5/6 nephrectomy model, in contrast to the Wang *et al* studies in diabetic kidneys, where miR-192 was decreased. The observed differences are likely to be related to the different model system used, and time points at which expression was assessed. Nevertheless, our data are consistent with the Kato *et al* and Chung *et al* studies. Furthermore, miR-192 is considered critical in downstream of TGF-β/Smad3 signalling in tubular epithelial cells 46. By using ChIP assays, we demonstrated that an antibody directed against Smad3 could successfully immunoprecipitate DNA fragments containing the potential Smad3-binding sites, thus supporting the Chung *et al* 46 findings that Smad3 could physically interact with the promoter region of miR-192, and that Taxol is capable of suppressing Smad3-mediated miR-192 transcriptional activity. To test our hypothesis that Taxol inhibits renal fibrosis through reduction of miR-192 levels, which down-regulate ECM protein expression, miRNA *in vitro* experiments were carried out. As expected, the tubular epithelial cells over-expressing miR-192 mimic or treated by TGF-β1 had significantly increased expression of not only miR-192 but also COL(I)A1 and COL(IV)A2. On the other hand, transfection of antisense miR-192 reduced the gene and protein expression induced by TGF-β1. These data indicate that miR-192 and ILK may have independent actions that converge at the downstream events related to TGF-β/Smad3 signalling in the development of renal fibrosis. The *in vitro* observations that miR-192 increases collagen expression, which is down-regulated by Taxol and also in the renal cortices of rat remnant kidney *in vivo*, underscores the significance of miR-192 among the three miRs in the pathobiology of interstitial fibrosis in this model.

In conclusion, low-dose Taxol significantly improves kidney functions and attenuates renal injury following subtotal renal ablation in rats by modulating mir-192 biology, which seems to be intimately interlinked with TGF-β/Smad signalling.

## Abbreviations

BUN: blood urea nitrogen
ECM: extracellular matrix
ILK: integrin-linked kinase
LNA: locked nucleic acid
PBS: phosphate buffer saline
TGF-β1: transforming growth factor-β
α-SMA: α-smooth muscle actin
TRT–PCR: real-time polymerase chain reaction
UUO: unilateral ureteral obstruction.
